# Small Nucleolar RNAs in Pseudoexfoliation Glaucoma

**DOI:** 10.3390/cells11172738

**Published:** 2022-09-02

**Authors:** Karolina Gasińska, Marcin Czop, Ewa Kosior-Jarecka, Dominika Wróbel-Dudzińska, Janusz Kocki, Tomasz Żarnowski

**Affiliations:** 1 Department of Diagnostics and Microsurgery of Glaucoma, Medical University of Lublin, 20-079 Lublin, Poland; 2 Department of Clinical Genetics, Medical University of Lublin, 20-080 Lublin, Poland

**Keywords:** small nucleolar RNA, snoRNA, aqueous humor, pseudoexfoliation glaucoma, PEXG, pseudoexfoliation syndrome, PEX

## Abstract

Small nucleolar RNAs (snoRNAs) are small non-coding regulatory RNAs that have been investigated extensively in recent years. However, the relationship between snoRNA and glaucoma is still unknown. This study aims to analyze the levels of snoRNA expression in the aqueous humor (AH) of patients with pseudoexfoliation glaucoma (PEXG) compared to a control group and identify hypothetical snoRNA-dependent mechanisms contributing to PEXG. The AH was obtained from eighteen Caucasian patients, comprising nine PEXG and nine age-matched control patients. RNA was isolated, and a microarray system was used to determine the snoRNA expression profiles. Functional and enrichment analyses were performed. We identified seven snoRNAs, *SNORD73B, SNORD58A, SNORD56, SNORA77, SNORA72, SNORA64*, and *SNORA32*, in the AH of the PEXG and control group patients. Five snoRNAs showed statistically significantly lower expression in the PEXG group, and two snoRNAs had statistically significantly higher expression in the PEXG group compared to the control group. In addition, we identified two factors—*CACNB3* for *SNORA64* and *TMEM63C* for *SNORA32*, similar to PEX-related genes (*CACNA1A* and *TMEM136*). The enrichment analysis for four genes targeted by snoRNAs revealed possible mechanisms associated with glaucoma and/or PEX, but the direct role of snoRNAs in these biological processes was not proven.

## 1. Introduction

Pseudoexfoliation syndrome (PEX) is an age-related systemic disease that affects 10–20% of the world’s population over the age of 60 [1]. The incidence rate varies geographically and ethnically. PEX is most common in Nordic, Baltic, Mediterranean, and Arab countries [2]. It is manifested by the deposition of abnormal gray–white material in eye tissues bathed by the aqueous humor (AH) and in the blood vessels, heart, lungs, liver, kidneys, and skin. PEX material is derived from the extracellular matrix (ECM) and contains elastic fibers composed of fibrillin-1, fibulin-2, vitronectin, lysyl oxidase, clusterin, and other proteins. Transforming growth factor beta 1 (TGF-β1) has been found to interact with lysyl oxidase in the formation of elastic fibers and is upregulated in anterior segment tissues in PEX [3]. Mechanisms leading to the development of PEX are complex and may include oxidative stress, hypoperfusion, and hypoxia [4]. PEX is associated with the development of pseudoexfoliation glaucoma (PEXG) and an increased risk of cardiovascular as well as cerebrovascular disorders [5]. In addition, Alzheimer’s disease, a progressive neurodegenerative condition in which extracellular amyloid plaques build up in the brain’s gray matter, is more common in PEX patients [6,7]. The evidence points to a strong genetic component of PEX. It is associated with single-nucleotide polymorphisms (SNPs) of numerous genes, including *LOXL1, FBLN5, CLU, GST, CNTNAP2, CACNA1A*, and *TMEM136* (also known as *TLCD5*) [3,4,5,6,7,8,9]. 

PEXG is the most common type of secondary glaucoma [10]. As with the other types of glaucoma, elevated intraocular pressure (IOP) is a major cause of retinal ganglion cell (RGC) apoptosis [11]. The key structural feature of the eye in the regulation of IOP is the human trabecular meshwork (HTM), which consists of trabecular cells surrounded by the ECM. The ECM has the greatest share in the production of resistance to AH outflow, and the disturbance of its homeostasis leads to an increase in IOP [12]. In PEXG, IOP often reaches high maximum values, and the large coexisting fluctuations in IOP also accelerate the progression of optic nerve degeneration [10,13]. However, as indicated in the long-term observations of patients, PEX material in the eyes does not always lead to increased IOP and glaucoma, or glaucoma occurs despite normal IOP [14,15]. This is evidence of complex mechanisms involved in the pathogenesis of PEXG and individually variable susceptibility to optic nerve damage. Therefore, the participation of genetic factors in determining these processes appears crucial. 

The glaucoma phenotype is determined not only by genes themselves but also by regulatory factors that influence the expression of these genes. The regulatory function may be performed by small non-coding RNA molecules, including microRNAs (miRNAs) and small nucleolar RNAs (snoRNAs). Many studies have uncovered extensive similarities between miRNA and snoRNA [16]. MiRNA is present in the AH, and its frequency and expression vary in different types of glaucoma [17]. However, there are no similar studies on snoRNA.

snoRNA molecules are some of the oldest in the evolution of non-protein-coding RNAs [18]. They are composed of 60–300 nucleotides and are located in the nucleolus—a nucleus region with high protein density [19,20]. A number of studies suggest that snoRNA localization to the nucleus is dynamic and regulated, and that it can relocalize to the cytoplasm [21]. Additionally, snoRNAs form specific complexes with proteins called small nucleolar ribonucleoproteins (snoRNPs). They are involved in the chemical modification of ribosomal RNA (rRNA) and small nuclear RNA (snRNA) nucleotides, and participate in rRNA maturation. SnoRNAs are classified into either C/D (SNORD) or H/ACA box (SNORA) subfamilies. C/D box snoRNAs catalyze 2′-O-ribose methylation and H/ACA box snoRNAs—pseudouridylation [22,23]. 

Emerging evidence demonstrates that snoRNAs are involved in various physiological and pathological cellular processes. Currently, only limited studies have reported the role of snoRNAs in ocular diseases. In an oxygen-induced proliferative retinopathy model in mouse retinas, 19 snoRNAs had significantly altered expression levels [24]. Another study on the expression profiles of snoRNAs in uveal melanoma (UM) showed 60 snoRNAs notably associated with the overall survival of UM patients [25]. Additionally, mutations and the aberrant expression of snoRNAs are reported in cell transformation, tumorigenesis, and metastasis, indicating that snoRNAs may serve as biomarkers and/or therapeutic targets of cancer [19]. For instance, by causing changes in ribosomal subunits (18S rRNA, 28S rRNA), snoRNAs are associated with various types of leukemia and cancers [26]. Additionally, a link between snoRNAs and Prader–Willi syndrome—a complex human neurological disease involving mental retardation, low height, obesity, and muscle hypotonia—has been found [27]. Furthermore, snoRNAs are reported to be differentially expressed in multiple sclerosis, a common inflammatory and degenerative disease that causes neurological disability [28]. 

As the relationship between snoRNA and glaucoma remains unclear, this study aimed to analyze the level of snoRNA expression in the AH of patients with PEXG compared to a control group as well as identify hypothetical snoRNA-dependent mechanisms contributing to PEXG.

## 2. Materials and Methods

### 2.1. Study Groups

In the present study, the AH was obtained from 18 Caucasian patients, comprising of nine PEXG and nine age-matched control patients (eight men and one woman in each group) who underwent routine cataract surgery at the Department of Diagnostics and Microsurgery of Glaucoma, Medical University of Lublin (Poland). All of the participants were over 18. Before the surgery, all of the patients underwent a comprehensive ophthalmic examination and medical history analysis. The ophthalmic examination included best-corrected visual acuity (BCVA) assessed with Snellen charts, an IOP test measured by Goldmann applanation tonometry (GAT), gonioscopy, a slit-lamp examination, indirect ophthalmoscopy after pupil dilation with a stereoscopic optic nerve head assessment, optical coherence tomography (Carl Zeiss Cirrus HD-OCT 5000), and standard automated perimetry (SAP) (24-2 strategy using the Humphrey perimeter). Two groups of patients were categorized, as are expanded upon below:

(I). Patients with incipient senile cataract and an advanced stage of PEXG: (a) cloudy area in the lens that causes decreased vision; (b) elevated IOP of greater than 21 mmHg, documented in medical history; (c) open-angle grade III/IV according to Schaffer’s classification; (d) glaucomatous optic nerve head damage (excavation, neuroretinal rim thinning or notching, and localized or diffuse retinal nerve fiber layer (RNFL) defect); (e) glaucomatous defect in SAP in at least two consecutive tests, with three reliability indices better than 15% (results were considered abnormal if the Glaucoma Hemifield Test result was outside normal limits and at least three contiguous points were present within the same hemifield on the pattern deviation (PD) plot at *p* < 1%, with at least one point at *p* < 0.5%); and (f) typical deposits of pathological material observed, during a slit-lamp examination, as concentric rings of grayish fringes on the peripheral lens capsule and flakes on the pupillary border of the iris.
(II). Control patients with incipient senile cataract: (a) cloudy area in the lens that causes decreased vision; (b) normal IOP (from 10 mmHg to 21 mmHg); and (c) no clinical signs of glaucoma nor PEX.

In all of the patients in Groups (I) and (II), other causes of RNFL thinning (i.e., myopia, optic disc anomalies, ischemic optic neuropathy, optic neuritis, hereditary optic neuropathy, traumatic optic neuropathy, multiple sclerosis, and degenerative diseases such as Alzheimer’s and Parkinson’s disease) were excluded after a careful medical history analysis, ophthalmologic examination, and OCT evaluation, including RNFL measurements and disc topography parameters. Moreover, all of the participants in Groups (I) and (II) had no previous intraocular surgeries, no other systemic or ocular diseases known to affect the visual field (e.g., pituitary lesions, demyelinating diseases, diabetes mellitus, etc.), and no previous eye or head trauma in their medical history.

The patients’ demographic and clinical data are presented in Table 1.

### 2.2. Aqueous Humor

AH samples (approximately 100 µL) were obtained from the eye’s anterior chamber at the beginning of the cataract surgery with special care to avoid contamination with blood or tears. 

### 2.3. RNA Isolation

The total RNA was isolated from the AH samples using an miRNeasy Serum/Plasma Kit (Qiagen, Valencia, CA, USA) according to the manufacturer’s instructions. The isolated RNA was stored at −80 °C for further analysis. The RNA concentration was determined by using a NanoDrop 2000c spectrophotometer (Thermo Fisher Scientific, Waltham, MA, USA). In addition, RNA analysis was performed using an Agilent Bioanalyzer 2100 (Agilent Technologies, Santa Clara, CA, USA) and a Pico RNA Kit according to the manufacturer’s protocol.

### 2.4. The snoRNA Profiling

A microarray system (GeneChip miRNA 4.0 Array chip, Affymetrix, Santa Carla, CA, USA) was used to determine the snoRNA expression profiles. The RNA preparation and hybridization were performed according to the manufacturer’s protocol, with one modification to extend the hybridization time to 42 h. The gene chips were scanned with an Affymetrix GeneChip Scanner 3000 (Affymetrix, Santa Carla, CA, USA).

The raw data, in a CEL format, were analyzed as log2-transformed intensities using the Affymetrix Transcriptome Analysis Console (TAC), following the software’s guidelines to determine differentially expressed genes (DEGs) between the PEXG and control patients. The snoRNAs that showed at least a one-fold difference between the control and PEXG groups, with a *p*-value of less than 0.01, were considered statistically significant and differentially expressed. 

### 2.5. Functional and Enrichment Analysis 

The data from TAC were used to create a snoRNA list. The analysis of the interactions of the selected snoRNAs with genes was carried out in the snoDB database [29]. The enrichment analysis was performed using the mirNET database, where a list of selected snoRNAs and a list of genes with which they interact were introduced [30]. Terms of the Kyoto Encyclopedia of Genes and Genomes (KEGG), REACTOME, and Gene Ontology (GO) categories were searched for in PEX-related pathways. 

### 2.6. Statistical Analysis

Graph Pad Prism 7 (Graph Pad Software, San Diego, CA, USA) and Statistica 13.3 (StatSoft, Krakow, Poland) were employed to prepare the Student’s *t*-test, chi^2^ test, and ROC curves. The distribution of the analyzed data was consistent with the normal distribution (Shapiro–Wilk test).

## 3. Results

Seven snoRNAs were detected as DEGs in the AH of the 18 Caucasian patients from the PEXG and control groups—*SNORD73B, SNORD58A, SNORD56, SNORA77, SNORA72, SNORA64,* and *SNORA32*. The list of detected snoRNAs along with their interactions are provided in Table 2. 

In the aqueous humor, we detected three C/D box snoRNAs (*SNORD73B, SNORD58A,* and *SNORD56*) and four H/ACA box snoRNAs (*SNORA77, SNORA72, SNORA64*, and *SNORA32*). Our analysis shows that snoRNAs interact with genes and other snoRNAs. These interactions are complex, and a single snoRNA molecule can act on many targets. For the seven selected snoRNAs, we found four target genes and three target snoRNAs. The above-mentioned interactions between snoRNAs and protein genes have been previously suggested in studies: Aw et al. (2016), Kretz et al. (2013), and Lu et al. (2016) [31,32,33]. SnoRNA host genes mainly encode proteins with a broad spectrum of biological roles, and three of them are RNA genes (AC012501.2, LINC02463, and PLS3-AS1) (Table 2).

We revealed target genes for two out of the seven snoRNAs. The SNORD56 molecule affects *FAM120AOS* (in the 3′UTR region), which encodes uncharacterized protein FAM120AOS (family with sequence similarity 120A opposite strand). The SNORA64 molecule regulates *RPS2* (interaction in the coding sequence of the gene) encoding ribosomal protein S2, which is a component of the 40S subunit, and also *TNPO2* (transportin-2) (interaction in the 3′UTR region) as well as *CACNB3* (interaction in the coding sequence of the gene) (regulatory subunit beta-3 of the voltage-dependent calcium channel) (Table 2 and Table 3).

The SNORD56 molecule interacts with the SNORA7B molecule, and their common host gene is *NOP56*. The second interaction is between SNORD56 and SNORA65 or SNORD50A molecules, and their common host gene is *SGSM1* (Table 2). 

**Figure 1:**
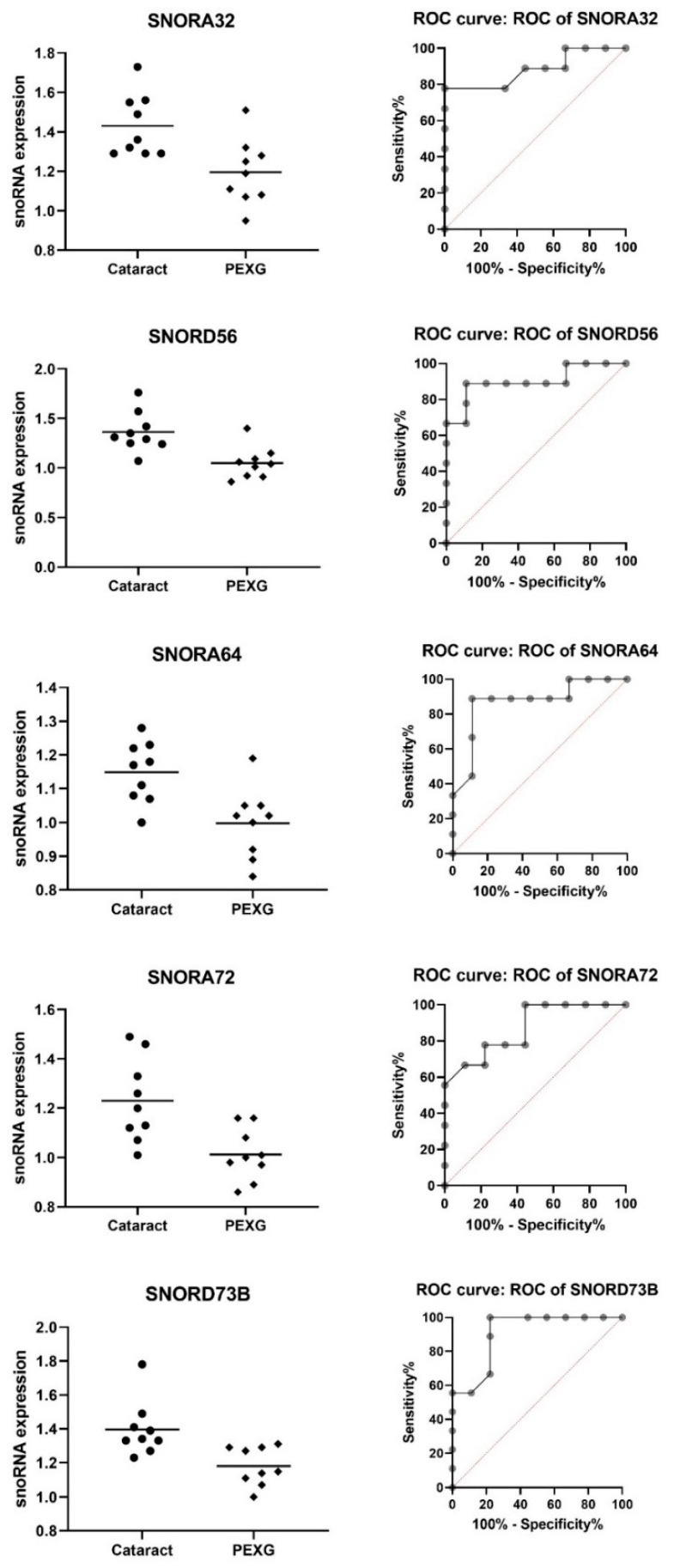
Lower snoRNA expression in the PEXG group compared to the cataract group. Data are shown as mean [log2].

By analyzing the results of snoRNA expression [log2], we found that five out of the seven snoRNAs—*SNORA32, SNORD56, SNORA64, SNORA72*, and *SNORD73B*—had lower expression in the PEXG group compared to the cataract group (*p*-value < 0.01; AUC ≥ 0.870; and AUC *p*-value < 0.01). *SNORA32* was 16.42% lower (0.235), *SNORD56* was 22.98% lower (0.313), *SNORA64* was 13.14% lower (0.151), *SNORA72* was 17.72% lower (0.218), and *SNORD73B* was 15.46% lower (0.216). The mean expression for all of the snoRNAs was 1.3138 in the cataract group and 1.0872 in the PEXG group, i.e., the mean decrease in expression level was 17.25% (0.2266). These data are shown in Table 4 and Figure 1. 

**Figure 2:**
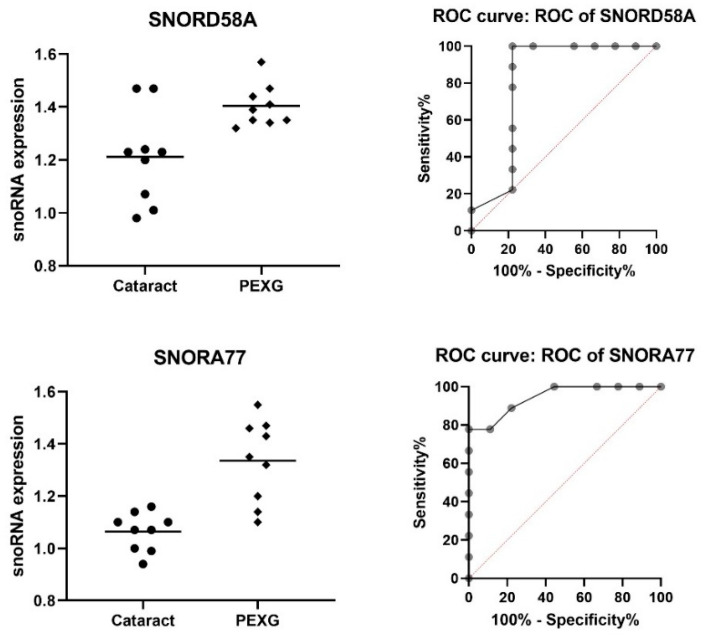
Higher snoRNA expression in the PEXG group compared to the cataract group. Data are shown as mean [log2].

Two out of the seven snoRNAs—*SNORD58A* and *SNORA77*—had a higher expression level in the PEXG group compared to the cataract group (*p*-value < 0.01; AUC ≥ 0.815; and AUC *p*-value < 0.03). *SNORD58A* was 15.94% higher (0.193), and *SNORA77* was 25.68% higher (0.273). The mean expression for all of the snoRNAs was 1.137 in the cataract group and 1.370 in the PEXG group, i.e., the mean increase in expression level was 20.49% (0.233). These data are shown in Table 5 and Figure 2.

**Figure 3:**
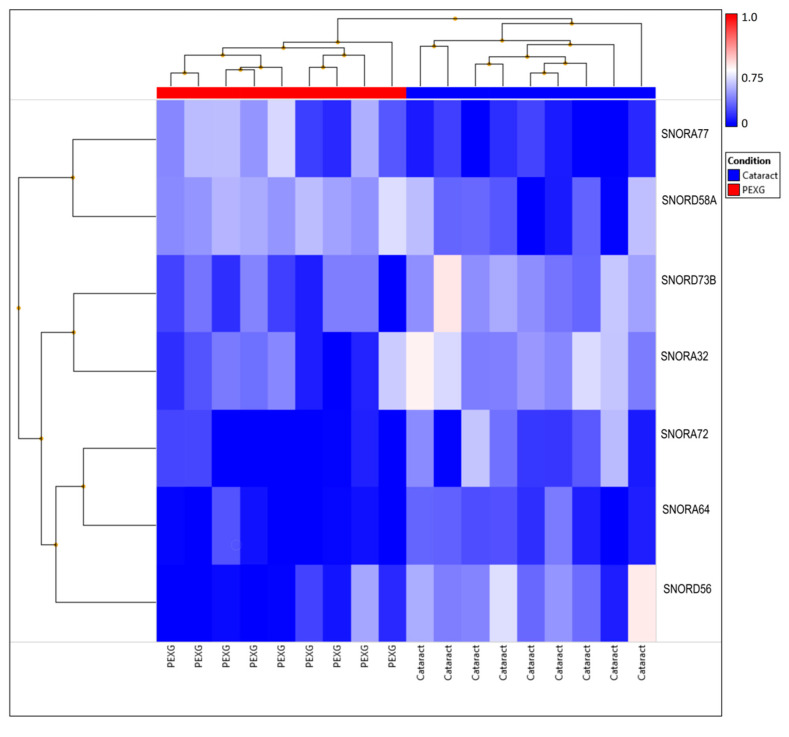
Cluster analysis results based on the expression levels of the detected snoRNAs.

We performed a cluster analysis based on the expression levels of the detected snoRNAs; the results are presented in Figure 3.

**Figure 4:**
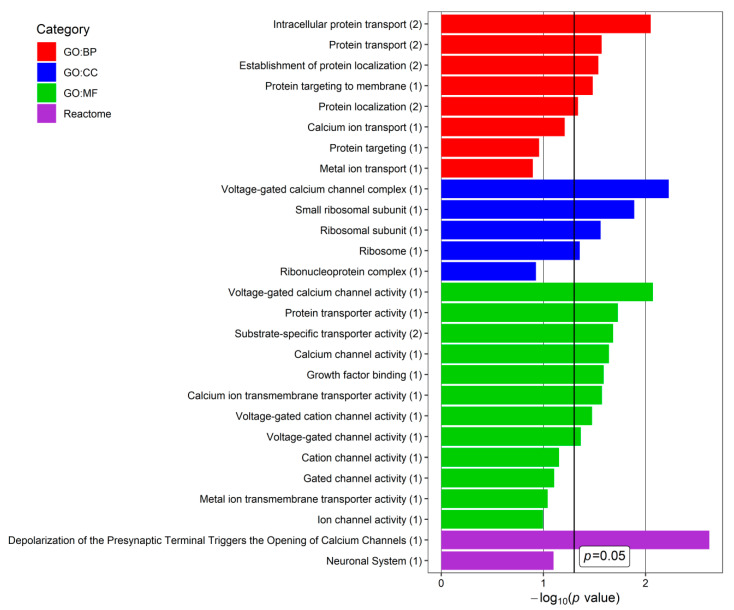
Terms of the Gene Ontology (GO): Biological Processing (GO:BP), GO: Cellular Compartment (GO:CC), GO: Molecular Function (GO:MF), and REACTOME categories, revealed for glaucoma- and/or pseudoexfoliation syndrome (PEX)-associated genes targeted by snoRNAs found in the current study in the aqueous humor (AH) of patients with PEXG and age-matched controls. *p*-value—EASE score for enrichment adjusted by the Benjamini correction for multiple-hypothesis testing. The number in brackets following the names of terms indicates the number of associated genes. The plot was generated using the ggplot2 3.3.0 package in R.

To indicate biological processes involving snoRNA-regulated genes, an enrichment analysis was carried out using the mirNET database for four genes: *CACNA3*, *FAM120AOS, RPS2,* and *TNPO2*. We selected four categories: GO: Biological Processing (GO:BP), GO: Cellular Compartment (GO:CC), GO: Molecular Function (GO:MF), and REACTOME. Genes indicated as targets of the expressed snoRNAs were associated with intracellular and extracellular structures in addition to various processes inside and outside cells. Selected data that may be potentially related to glaucoma and/or PEX are presented in Figure 4. In GO:BP, we selected eight terms; in GO:CC, five; in GO:MF, twelve; and in REACTOME, two, i.e., twenty-seven terms in total. The most significant glaucoma- and/or PEX-associated terms concerned the ion signaling pathway (especially calcium) and protein transport. Explanations of terms of the GO:BP, GO:CC, GO:MF, and REACTOME categories are presented in Table S1.

## 4. Discussion

Although snoRNAs have been known since the late 1960s, and the term “snoRNA” has been used since 1981 [34], their involvement in physiological and pathological processes remains poorly understood. This may be due to a considerable diversity in non-coding RNAs, which interact with one another and together regulate gene expression. A large proportion of snoRNAs are further processed into smaller molecules, some of which display different functionalities. Comparing the genomic locations of snoRNAs and miRNAs reveals an overlap of specific members of these classes. Both groups have members encoded in introns and others encoded in independent transcription units. Furthermore, the proportion of intronic snoRNAs generally correlates with the proportion of intronic miRNAs. In addition, there are similarities in the processing pathways of snoRNAs and miRNAs [16,35].

Several protein-coding host genes encode both snoRNAs and miRNAs in distinct introns. For example, *NOP56* (*locus 20p13*), found as the target gene for *SNORD56*, detected in our study, encodes the box C/D snoRNAs *SNORD56, SNORD57, SNORD86,* and *SNORD110*, the box H/ACA snoRNA *SNORA51*, and the miRNA miR-1292. Additionally, *HTR2C* (*locus Xq23*), which has a similar *locus* as *PLS3-AS1* (*locus Xq23*), found as the target gene for *SNORA64*, detected in our study, encodes the box H/ACA snoRNA *SNORA35* as well as the miRNAs miR-448, miR-1264, miR-1298, miR-1911, and miR-1912 [16].

One study shows almost 400 different snoRNAs in the murine retina and RPE/choroid. *SNORD27* was the most abundant in the retina, while *SNORD58* was in the RPE/choroid (85-fold higher than in the retina). In addition, snoRNAs can be processed to generate snoRNA-derived small RNAs that resemble microRNAs (sno-miRNAs). Many of these molecules have also been detected in the retina and RPE/choroid [36].

The role of snoRNAs is crucial in genetic diseases because it can lead to a better understanding of their underlying mechanisms and establish potential RNA-based biomarkers in addition to future therapies. One of the most important eye diseases with a genetic background is glaucoma—a common cause of blindness worldwide. PEXG is a type of secondary open-angle glaucoma that accompanies PEX, a systemic disease manifesting primarily in the eyes via the accumulation of pathological material in the anterior segment. The prognosis of PEXG is often worse than that of primary open-angle glaucoma (POAG), wherein patients may present with a more severe clinical course, a poorer response to medications, and a more frequent necessity for surgical intervention. The mechanisms leading to the development of glaucomatous neuropathy are complex and not fully understood. The main risk factor is increased IOP caused by the impaired outflow of AH from the anterior chamber through the HTM [13]. Oxidative stress occurring in the anterior chamber damages the HTM, contributing to an increase in IOP, and in the retina may directly cause the apoptosis of RGCs. Moreover, ocular blood supply disorders play an important role [37]. Additionally, the potential genetical tendency of RGCs for excessive apoptosis is also kept in mind.

Some researchers investigated the role of small nucleolar RNA host gene 3 (*SNHG3*, *locus 1p35.3*) in HTM cells under oxidative stress. Hydrogen peroxide (H_2_O_2_) induces an acute oxidative stress injury in HTM cells to create a glaucoma model. They observed H_2_O_2_-induced SNHG3 upregulation in HTM cells. SNHG3 silencing alleviated H_2_O_2_-induced oxidative damage in HTM cells. Moreover, SNHG3 cooperated with ELAV-like RNA-binding protein 2 (ELAVL2) to modulate cell apoptosis and ECM accumulation in HTM cells under oxidative stress [38]. Similar findings have been reported in another study using a model of H_2_O_2_-induced oxidative stress in HTM cells. Oxidative stress conditions in HTM cells significantly upregulated SNORD3A [39]. 

Furthermore, one study investigated snoRNA expression in developing mouse lenses. As a result, 167 snoRNAs were found in total—113 C/D box snoRNAs and 54 H/ACA box snoRNAs. Notably, the upregulation of snoRNAs in the postnatal mouse lens transcriptome was observed [40]. In our study, we chose patients with incipient senile cataract without other ocular diseases as a control group; however, the impact of cataract on the snoRNA expression results in the PEXG group should be taken into consideration. As the role of snoRNA-dependent pathways in cataract pathogenesis is currently unknown, this is a major limitation of our study. On the other hand, there is no ethically approved possibility to obtain the AH from healthy individuals, and senile cataract appears in almost every older patient at different stages. 

Our work demonstrated that in the AH of patients with PEXG and control cataract, seven snoRNAs, *SNORD73B, SNORD58A, SNORD56, SNORA77, SNORA72, SNORA64*, and *SNORA32*, can be identified, including statistically significant differences in expression levels. Five snoRNAs, *SNORA32, SNORD56, SNORA64, SNORA72*, and *SNORD73B*, had lower expression in the PEXG group, and two snoRNAs, *SNORD58A* and *SNORA77*, had higher expression in the PEXG group compared to the cataract group. The differences in snoRNA expression were small; therefore, based on this information, we cannot conclude that they have a measurable effect on the function of their targets. Moreover, patients in our study had advanced PEXG, which suggests that snoRNA molecules may be the result of the disease process and not a causative factor, and they cannot be considered as biomarkers. A comparison of snoRNAs in the early stages of PEXG is needed.

The study that we conducted is the first attempt to discover the types of snoRNA present in the AH of patients with PEXG and PEX. Additionally, there are no studies showing snoRNA profiles in the AH in any other diseases. The origin of snoRNA in the AH is not clear. They may be derived from exosomes that are a major constituent of AH. Exosomes function in extracellular communication and contain proteins as well as small RNA [41]. Another reason for the presence of snoRNA in the AH may be surgical procedures that cause blood–aqueous barrier disruption with the leakage of cells in the anterior chamber [42]. snoRNA could also be released from damaged corneal endothelium cells. The AH is a unique material, available in small amounts, and can only be obtained intraoperatively.

The snoRNAs identified in our study have not been described in other ocular diseases. In one study, *SNORD58* was detected in the RPE/choroid at a very high expression level. The exact function of this snoRNA is unknown, but the most highly expressed sno-miRNAs in this localization are implicated in the maintenance of the blood–retinal barrier and in promoting RPE differentiation [36]. In diseases not related to the eyes, *SNORD56* was upregulated in uterine leiomyoma [43]. Additionally, *SNORA72* was found to be highly expressed in ovarian sphere cells. The overexpression of *SNORA72* increased ovarian cancer cells’ self-renewal and migration abilities [44]. Furthermore, *SNORA64* expression was increased in multiple myeloma, prostate cancer, and X-linked dyskeratosis congenita [26]. 

Our study identified four target genes and three target snoRNAs for the investigated snoRNA molecules. None of these findings have previously been associated with PEXG and PEX. However, *CACNB3* (targeted by *SNORA64*) is similar to a PEX-associated gene—*CACNA1A*.

Calcium channels play an important role in PEX pathogenesis because a high calcium concentration in direct association with aggregating PEX fibrils was detected. One of the components of PEX material is fibrillin, which utilizes calcium to form stable aggregates; therefore, the altered function of a calcium channel could facilitate the formation of PEX aggregates [8]. Calcium channels are responsible for the transport of calcium ions across the cell membrane. They are multisubunit complexes composed of alpha-1, beta, and alpha-2/delta subunits. The channel activity is directed by the pore-forming alpha-1 subunit, whereas the others act as auxiliary subunits regulating this activity [45]. The *CACNA1A* (*locus 19p13.13*) encodes the alpha-1A subunit of the voltage-gated calcium channel and is expressed in the eye in the anterior lens epithelium, iris, ciliary body, optic nerve glia, and vascular endothelial cells [8]. *CACNB3* (*locus 12q13.12*) encodes the regulatory subunit beta-3 of the voltage-gated calcium channel. Beta subunits are composed of five domains, which contribute to the regulation of surface expression and the gating of calcium channels, and may also regulate gene transcription [46,47,48]. It has been proven that calcium channels may participate in PEX material formation by increasing the calcium concentration [8]. Moreover, one of the studies showed that the visual pathway development is disrupted in mice with a targeted disruption of the *CACNB3* gene [49]. In a rat model of cerebral ischemia, *CACNB3*, which might represent functional modules within the ischemic neuronal transcriptome, was induced [50]. In bipolar disorder, the elevation of miR-34a expression in human neuronal progenitors affected *CACNB3* mRNA and protein expression, and resulted in defects in neuronal differentiation [51]. 

As the host genes of snoRNAs have been shown to be abnormally expressed in multiple cancers [52], they could also have an impact on other diseases. In Table 2, we presented the *SNORA32* host gene, *TMEM63C*, which shows similarity to the *TMEM136* gene described in PEX.

A pronounced and early vasculopathy, partly involving PEX material deposits around ocular blood vessels, appears to play a significant role in PEX [9]. Vasculopathy is caused by the dysregulation of endothelial cell function within the vascular wall, which leads to vascular remodeling [53]. Cell stabilization is provided by transmembrane proteins, which participate in the transport of ions and other substances across the cell membrane and are the basis of most cell surface receptors [54]. *TMEM136* (*locus 11q23.3*) encodes transmembrane protein 136, which was found to be primarily localized to the endothelial cells of blood vessels and aqueous outflow structures, and may contribute to vascular and trabecular dysfunction in PEX eyes. The role of TMEM136 remains unclear, but it seems to maintain the proper functioning of cells by regulating cellular potassium ion transport. Some studies have shown significantly reduced expression of TMEM136 in iris and ciliary body tissues in PEX patients [3,9,55,56]. *TMEM63C* (*locus 14q24.3*) encodes transmembrane protein 63C, which enables calcium-activated cation channel activity and is involved in cation transport [57,58]. Calcium participates in regulating voltage-gated sodium, calcium, and potassium channels [59]. Calcium and potassium channels may be relevant in PEX development [3,8]. Recent studies show the possible role of transient receptor potential (TRP) channels in glaucoma. They are a cluster of non-selective cation channels present on cell membranes. Various TRP subfamilies are abundantly expressed in ocular structures, including the cornea, lens, ciliary body, HTM, and retina. They are involved in AH secretion and cytoskeleton remodeling of the HTM to control IOP by affecting retinal function [60]. However, there is no clear evidence for the involvement of TRP channels in the pathogenesis of PEX. In a study on age-related macular degeneration, a suggestive association signal in *TMEM63C* was found, but its role in AMD pathogenesis is unknown [61]. Additionally, *TMEM63C* is considered a biomarker of focal segmental glomerulosclerosis (FSGS) [57,62]. FSGS shows the obliteration or collapse of glomerular capillary loops via increased ECM [63]. Similar changes in blood vessels are observed in PEX patients, as PEX is frequently linked to increased vascular resistance and decreased blood flow velocity, arterial endothelial dysfunction, and arterial hypertension. This could imply that PEX and FSGS have common features in their pathogenesis [5].

To reveal the predicted associations with the biological processes, we performed enrichment analyses for four genes, *CACNA3*, *FAM120AOS, RPS2,* and *TNPO2*, targeted by *SNORA32, SNORA64, SNORA72, SNORA77, SNORD58A,* and *SNORD73B*, using the mirNET database. However, based on these data, we cannot prove that actual interactions between snoRNAs and their predicted targets exist because of a lack of experimental evidence for such correlations. On the other hand, these interactions have been suggested in previous studies: Aw et al. (2016), Kretz et al. (2013), and Lu et al. (2016) [31,32,33]. Terms associated with PEX pathogenesis include protein transport, protein localization, protein targeting to membranes, ribosomes (ribonucleoprotein complexes responsible for protein synthesis in cells), calcium channel activity, and growth factor binding. Simultaneously, impaired protein expression, the dysfunction of calcium channels, and TGF accumulation are well-known as glaucoma-associated factors. 

During the onset of glaucoma, the elevation in IOP causes ischemia, hypoxia, and oxidative stress accumulation in the retina, all of which contribute to the apoptosis of RGCs [60]; AH outflow resistance is determined in particular by the ECM [64]. Along with increased protein deposition (mainly collagen and elastin), the ECM becomes more dense and stiff, which leads to an increase in IOP [65]. Many ECM-stimulating factors are found to be upregulated in glaucoma, including TGF-β2 in the AH and actin secreted by trabecular cells [65,66]. Additionally, IOP is regulated by HTM cell contraction (nitric oxide) [67], the cytoskeleton density of trabecular and Schlemm’s canal cells (RhoA protein) [68,69], and by transmembrane proteins that ensure the calcium balance as well as participate in the adhesion of these cells (integrins) [70]. Increased IOP results in retinal blood flow disorder and, therefore, antioxidant imbalance, leading to the death of RGCs [71]. 

## 5. Conclusions

The study that we conducted is the first attempt to discover the types of snoRNA present in the AH of patients with PEXG and PEX. We identified seven snoRNAs: five snoRNAs, *SNORA32, SNORD56, SNORA64, SNORA72*, and *SNORD73B*, had statistically significant lower expression in the PEXG group (mean decrease of 17.25%), and two snoRNAs, *SNORD58A* and *SNORA77*, showed statistically significant higher expression in the PEXG group (mean increase of 20.49%) compared to the control group. However, this small change in snoRNA expression may not be functionally significant.

Moreover, we found two factors, the *CACNB3* target gene for *SNORA64* and the *TMEM63C* host gene for *SNORA32*, that could be potentially important in PEX pathogenesis, as they have similar functions to the *CACNA1A* and *TMEM136* genes in PEX. In addition, the enrichment analysis for four genes targeted by snoRNAs revealed possible mechanisms associated with glaucoma and/or PEX, but the direct role of snoRNAs in these biological processes is not proven. Further research is needed to better understand the functional diversity of snoRNA molecules and their role in PEXG.

## Figures and Tables

**Table 1:** Demographic and clinical characteristics of the study groups.

		**PEXG** **N = 9**	**Cataract** **N = 9**	***p*** **-Value**
Age (mean ± SD)		77.56 ± 6.54	76.00 ± 6.95	0.6314 ^^^
Gender (N, %)	Male	8 (88.89%)	8 (88.89%)	0.7647 ^#^
	Female	1 (11.11%)	1 (11.11%)	
BCVA		0.19 ± 0.17	0.42 ± 0.12	0.0047 ^^^
MAX IOP		30.44 ± 13.1	15.67 ± 2.12	0.0042 ^^^
C/D		0.91 ± 0.07	0.29 ± 0.15	<0.0001 ^^^
RNFL		61.22 ± 5.12	93.8 ± 5.61	0.0005 ^^^
MD		−22.55 ± 4.77	−0.03 ± 0.98	<0.0001 ^^^

^#^—chi^2^ test; ^^^—Student’s *t*-test; BCVA—best-corrected visual acuity; C/D—cup/disc; IOP—intraocular pressure; MD—mean deviation; PEXG—pseudoexfoliation glaucoma; RNFL—retinal nerve fiber layer; and SD—standard deviation.

**Table 2:** List of the snoRNAs examined in the study and interactions with genes and snoRNAs.

Symbol	Box Type	Host Genes	Target Genes	Target snoRNAs
SNORD73B	C/D	*RPS3A*	-	-
SNORD58A	C/D	*RPL17*	-	-
SNORD56	C/D	*EDEM2*	-	-
		*LINC02463*	-	-
		*AC012501.2*	-	-
		*ITGB8*	-	-
		*NOP56*	-	SNORA7B
		*SGSM1*	-	SNORA65, SNORD50A
		*-*	*FAM120AOS*	-
SNORA77	H/ACA	*RERE*	-	-
		*ATP2B4 OR ATP2B2*		
SNORA72	H/ACA	*RPL30*	-	-
		*NUCKS1*	-	-
		*ECT2*	-	-
		*DEGS1*	-	-
SNORA64	H/ACA	*MYRIP*	-	-
		*RPS2*	*RPS2, TNPO2*	-
		*PLS3-AS1*	-	-
		*-*	*CACNB3*	-
SNORA32	H/ACA	*TAF1D*	-	-
		*TMEM63C*	-	-

snoRNAs—small nucleolar RNAs.

**Table 3:** Features of genes regulated by snoRNAs examined in the study.

Symbol	Chromosome	Description
*CACNB3*	*12q13.12*	Calcium voltage-gated channel auxiliary subunit beta 3
*FAM120AOS*	*9q22.31*	Family with sequence similarity 120A opposite strand
*RPS2*	*16p13.3*	Ribosomal protein S2
*TNPO2*	*19p13.13*	Transportin 2

**Table 4:** Lower snoRNAs’ expression in the PEXG group compared to the cataract group [log2].

snoRNA	Group	N	Mean	SD	Fold Change	*p*-Value	AUC	AUC *p*-Value
SNORA32	Cataract	9	1.431	0.158	−1.14	0.0072	0.883	0.0062
	PEXG	9	1.196	0.166				
SNORA72	Cataract	9	1.230	0.169	−1.15	0.0047	0.870	0.0081
	PEXG	9	1.012	0.106				
SNORD56	Cataract	9	1.362	0.202	−1.21	0.0022	0.901	0.0041
	PEXG	9	1.049	0.161				
SNORA64	Cataract	9	1.149	0.090	−1.10	0.0045	0.870	0.0081
	PEXG	9	0.998	0.104				
SNORD73b	Cataract	9	1.397	0.163	−1.12	0.0048	0.907	0.0036
	PEXG	9	1.181	0.112				

AUC—area under the curve.

**Table 5:** Higher snoRNA expression in the PEXG group compared to the cataract group [log2].

snoRNA	Group	N	Mean	SD	Fold Change	*p*-Value	AUC	AUC *p*-Value
SNORD58A	Cataract	9	1.211	0.177	1.13	0.0086	0.815	0.0243
	PEXG	9	1.404	0.079				
SNORA77	Cataract	9	1.063	0.073	1.21	0.0003	0.944	0.0015
	PEXG	9	1.336	0.159				

## Data Availability

All data generated or analyzed during this study are included in this published article (and its Supporting Information File).

## Supplementary Materials

The following supporting information can be downloaded at: https://www.mdpi.com/article/10.3390/cells11172738/s1, Table S1: Explanations of terms of the Gene Ontology (GO): Biological Processing (GO:BP), GO: Cellular Compartment (GO:CC), GO: Molecular Function (GO:MF), and REACTOME categories.
