# Supplementary material for: Small Nucleolar RNAs in Pseudoexfoliation Glaucoma
Source: Cells. 2022 Sep 2;11(17):2738. doi: 10.3390/cells11172738 (PMC9454646; doi:10.3390/cells11172738)
Supplement: Supplementary file 1 [file cells-11-02738-s001.zip › cells-1854268-supplementary.pdf]

Table S1. Explanations of terms of the Gene Ontology (GO): Biological Processing (GO:BP), GO: Cellular Compartment (GO:CC), GO: Molecular Function (GO:MF), and REACTOME categories.

Category	Term	Definition
GO: Biological Processing	intracellular protein transport	The directed movement of proteins in a cell, including the movement of proteins between specific compartments or structures within a cell, such as organelles of a eukaryotic cell. less
	protein transport	The directed movement of proteins into, out of or within a cell, or between cells, by means of some agent such as a transporter or pore.
	establishment of protein localization	The directed movement of a protein to a specific location.
	protein targeting to membrane	The process of directing proteins towards a membrane, usually using signals contained within the protein.
	protein localization	Any process in which a protein is transported to, or maintained in, a specific location.
	calcium ion transport	The directed movement of calcium (Ca) ions into, out of or within a cell, or between cells, by means of some agent such as a transporter or pore.
	protein targeting	The process of targeting specific proteins to particular regions of the cell, typically membrane-bou more...
	metal ion transport	The directed movement of metal ions, any metal ion with an electric charge, into, out of or within a more...
GO: Cellular Compartment	voltage-gated calcium channel complex	A protein complex that forms a transmembrane channel through which calcium ions may pass in response to changes in membrane potential.
	small ribosomal subunit	The smaller of the two subunits of a ribosome.
	ribosomal subunit	Either of the two subunits of a ribosome: the ribosomal large subunit or the ribosomal small subunit.
	ribosome	An intracellular organelle, about 200 A in diameter, consisting of RNA and protein. It is the site o more...
	ribonucleoprotein complex	A macromolecular complex that contains both RNA and protein molecules.
GO: Molecular Function	voltage-gated anion channel activity	Enables the transmembrane transfer of an anion by a voltage-gated channel. An anion is a negatively charged ion. A voltage-gated channel is a channel whose open state is dependent on the voltage across the membrane in which it is embedded. less
	protein transporter activity	Directly binding to a specific protein and delivering it to a specific cellular location.
	substrate-specific transporter activity	Enables the transfer of a substance, usually a specific substance or a group of related substances, from one side of a membrane to the other.
	calcium channel activity	Enables the facilitated diffusion of a calcium ion (by an energy-independent process) involving passage through a transmembrane aqueous pore or channel without evidence for a carrier-mediated mechanism. less
	growth factor binding	Binding to a growth factor, proteins or polypeptides that stimulate a cell or organism to grow or proliferate.
	calcium ion transmembrane transporter activity	Enables the transfer of calcium (Ca) ions from one side of a membrane to the other.
	voltage-gated channel activity	Enables the transmembrane transfer of a solute by a channel whose open state is dependent on the voltage across the membrane in which it is embedded.
	cation channel activity	Enables the energy-independent passage of cations across a lipid bilayer down a concentration gradient.
	gated channel activity	Enables the transmembrane transfer of a solute by a channel that opens in response to a specific stimulus.
	metal ion transmembrane transporter activity	Enables the transfer of metal ions from one side of a membrane to the other.
	ion channel activity	Enables the facilitated diffusion of an ion (by an energy-independent process) by passage through a transmembrane aqueous pore or channel without evidence for a carrier-mediated mechanism. May be either selective (it enables passage of a specific ion only) or non-selective (it enables passage of two or more ions of same charge but different size). less
REACTOME	depolarization of the presynaptic terminal triggers the opening of calcium channel	Action potentials occur in electrically excitable cells such as neurons, muscles, and endocrine cells. They are initiated by transient opening of voltage dependent sodium channels, causing a rapid, large depolarization of membrane potentials that spread along the axon membrane. The action potential travels down the axon and reaches the presynaptic terminal depolarizing the membrane in the pre synaptic terminal. The depolarization causes the voltage gated Ca2+ channels to open allowing the influx of Ca2+ that signals the release of neurotransmitter into the synaptic cleft.
	neuronal system	The human brain contains at least 100 billion neurons, each with the ability to influence many other cells. Clearly, highly sophisticated and efficient mechanisms are needed to enable communication among this astronomical number of elements. This communication occurs across synapses, the functional connection between neurons. Synapses can be divided into two general classes: electrical synapses and chemical synapses. Electrical synapses permit direct, passive flow of electrical current from one neuron to another. The current flows through gap junctions, specialized membrane channels that connect the two cells. Chemical synapses enable cell-to-cell communication using neurotransmitter release. Neurotransmitters are chemical agents released by presynaptic neurons that trigger a secondary current flow in postsynaptic neurons by activating specific receptor molecules. Neurotransmitter secretion is triggered by the influx of Ca2+ through voltage-gated channels, which gives rise to a transient increase in Ca2+ concentration within the presynaptic terminal. The rise in Ca2+ concentration causes synaptic vesicles (the presynaptic organelles that store neurotransmitters) to fuse with the presynaptic plasma membrane and release their contents into the space between the pre- and postsynaptic cells.
